# Advances in Development, Characterisation and Application of Nasal Drug Delivery Systems

**DOI:** 10.3390/pharmaceutics14081562

**Published:** 2022-07-27

**Authors:** Anita Hafner

**Affiliations:** University of Zagreb, Faculty of Pharmacy and Biochemistry, Department of Pharmaceutical Technology, A. Kovačića 1, 10000 Zagreb, Croatia; anita.hafner@pharma.unizg.hr

Nasal drug administration is being extensively investigated for local and systemic drug delivery, brain targeting and mucosal vaccination. Innovative nasal drug delivery systems present the key strategy to increase drug availability at the site of action, adjusting its dissolution properties and permeation profile to the treated condition, and promoting its retention at the nasal mucosa. Furthermore, well-designed drug delivery systems reduce possible side-effects and improve patient compliance, leading to improved therapy outcomes. The anatomy and physiology of the nasal cavity and/or nasal mucosa are recognised as the most critical factors in nasal drug delivery, among which is the mucocilliary clearance that shortens the formulation residence time at the nasal mucosa, and complex nasal geometry that hinders formulation delivery to the targeted regions of the nasal cavity. These obstacles are clearly related to the need for the advancement and implementation of in vitro/ex vivo characterisation methods for early prediction of therapeutic potential of the nasal delivery system and screening regarding the efficiency of delivery to distinct regions of the nasal cavity.

This Special Issue, therefore, highlights the latest strategies and approaches in development, characterisation and application of nasal drug delivery systems.

Diverse formulation approaches in nasal drug delivery have been presented, including development of binary composite solid microparticles [1], nanovesicular systems [2] and microsphere/inert carrier dry powder platform [3], all designed for direct nose-to-brain delivery of the incorporated drug.

Vasa et al. [1] developed binary composite solid microparticles of the antiviral drug ribavirin and poloxamer as a nasal permeation enhancer. The preparation method included the suspension of fine crystals of ribavirin in a molten matrix of poloxamer, quenching using liquid nitrogen and cryogenic milling. The prepared microparticles were characterised by appropriate drug content uniformity, as well as particle size and drug release properties suited for nasal delivery. In vitro evaluation of the composite solid microparticles showed improved ribavirin permeation across excised olfactory mucosa compared to solid drug and ribavirin aqueous solution, indicating improved potential for ribavirin direct nose-to-brain delivery.

Touitou et al. [2] designed propylene glycol-comprising soft nanovesicles, as an efficient nasal delivery system of analgesics. Pharmacokinetic studies of nasally administered tramadol nanovesicular systems in rats revealed a rapid increase in plasma and brain drug concentrations and the maximum drug concentration (*C*_max_) was reached 10 min after nasal administration, which was 2 to 5 folds greater than the *C*_max_ reached upon oral or nasal non-vesicular drug delivery, respectively. Similarly, nasal administration of ketoprofen nanovesicular systems resulted in fast drug absorption and the plasma *C*_max_ was reached 10 min after administration, which was three times higher than that after oral administration. The analgesic effect of each nanovesicle-incorporated drug, ketoprofen, butorphanol or tramadol, was found to be rapid and improved when compared to that of oral and nasal controls, as assessed in the acetic acid mice model for pain. The investigated approach presents a promising strategy for improved and non-invasive pain treatment.

The nasal route of administration has been recently proposed for glucocorticoid brain-targeted delivery in the treatment of neuroinflammation processes observed in patients with severe COVID-19. Nižić Nodilo et al. [3] addressed this issue with the development of a sprayable brain-targeting powder delivery platform of dexamethasone sodium phosphate (DSP), consisting of DSP-loaded hypromellose- and pectin-based microspheres, blended with mannitol as an inert carrier. The QbD approach was applied for the rational design of spray-dried DSP-loaded microspheres. The optimised microspheres/mannitol powder blend was characterised by appropriate homogeneity, sprayability and biopharmaceutical properties. The experimental set-up included nasal deposition studies and evaluation of administration parameters in 3D-printed nasal cast, highlighting the importance of nasal deposition consideration in the early phase of formulation development. A relatively high DSP dose fraction was successfully delivered to the olfactory region, revealing the potential of the developed powder platform for brain-targeted delivery. Deposition studies also highlighted the importance of the individual approach when aiming for the olfactory region.

This Special Issue brought an insight into new or improved in vitro characterisation methods applicable in nasal formulation development.

Trenkel and Scherließ [4] developed simple and useful in vitro characterisation methods aimed at screening for the impact of powders on nasal residence time and sensory effects. The range of mucoadhesive polymers used revealed the applicability of rheological characterisation, combined with dynamic vapour sorption and adhesiveness on agar–mucin plates, for the comparative assessment of the potential to increase the nasal residence time. The effect was shown to be dependent on polymer chain length and charge, extent of vapour sorption and specific interactions with mucin. To screen for the sensory effects of close contact of powders with the mucosa, a slug mucosal irritation assay was adapted to the powders. Evaluating mucus production in slugs in contact with fillers and mucoadhesive agents allowed for a meaningful comparison of the different excipients. The obtained results suggested that nasal discomfort depended on the nasal powder particle size and charge. The described in vitro characterisation approaches can be applied to both excipients for nasal powders and nasal powder formulations, presenting a significant contribution to screening tools in the development of powders as promising nasal delivery platforms.

In vitro diffusion and dissolution tests present inevitable procedures in the development of nasal formulations; however, there are still no official methods for this type of assessment of nasal drug products. Therefore, Bartos et al. [5] assessed and compared the suitability of vertical diffusion cells (Franz cells) and horizontal diffusion cells (Side-Bi-Side) for in vitro evaluation of drug permeation across the nasal mucosa from different nasal formulations, including liquid (sprays), semisolid (gel) and solid (powder) formulations, as the official pharmacopoeial nasal dosage forms. The investigated formulations contained raw or nanonized meloxicam, a poorly water-soluble nonsteroidal anti-inflammatory drug that can be nasally applied for acute pain therapy or to enhance analgesia. The performed study confirmed the suitability of vertical cells for the evaluation of semi-solid formulations and revealed the potential for using horizontal cells in the assessment of liquid formulations and powders suspended in the phosphate buffer. The authors pointed out the advantages of horizontal diffusion cells, including the possibility of donor chamber stirring and elimination of the positive effect of gravitation on diffusion observed in vertical diffusion cells, but not in nasal conditions in vivo.

Cell models of the nasal epithelial barrier represent important in vitro screening tools in tailoring biocompatible and drug permeation enhancing nasal delivery system. Sibinovska et al. [6] studied the utilisation of RPMI 2650 and Calu-3 cell lines for the assessment of the formulations’ effect on drug permeability. Namely, cell model permeation of locally and systemically acting drugs formulated in different pharmaceutical dosage forms (solutions and suspensions) was evaluated. Both RPMI 2650 and Calu-3 cell models cultured at an air–liquid interface showed the potential to disclose the effect of formulation properties on drug permeability. The differences in cell model permeability profiles observed for the different formulations of the same drug corresponded well to the differences in bioavailability in vivo. The results obtained provided new perspectives on drug formulations in in vitro permeability studies and in vitro support to bioequivalence studies.

Finally, this Special Issue brings the thorough review by Pilicheva and Boyuklieva [7] on the current trends and investigations on nasal vaccines and drug delivery for combating COVID-19. The comprehensive presentation of the state-of-the-art research in this area highlights the wide range of nasal therapeutic options that are applicable in tackling the COVID-19 pandemic, including local and systemic drug delivery, nasal mucosal vaccination and brain targeting.

As a concluding remark, this Special Issue outlines the progress in diverse aspects of nasal drug delivery. Nonetheless, it contributes to opening new questions and perspectives and highlights the need for further investigations to boost the utilisation of versatile nasal drug delivery potential.
